# A novel method to establish glucocorticoid resistant acute lymphoblastic leukemia cell lines

**DOI:** 10.1186/s13046-019-1280-2

**Published:** 2019-06-20

**Authors:** Ling Gu, Ge Zhang, Yanle Zhang

**Affiliations:** 1 Laboratory of Hematology/Oncology, Department of Pediatric Hematology/Oncology, Key Laboratory of Birth Defects and Related Diseases of Women and Children (Sichuan University), Ministry of Education, West China Second University Hospital, Sichuan University, No.20, Section 3, Renmin South Road, Chengdu, 610041 People’s Republic of China; 2 Joint laboratory of West China Second University Hospital, Sichuan University and School of Life Science, Fudan University for Pulmonary Development and Disease, Chengdu, China; 3 Department of Laboratory Medicine, West China Second University Hospital, Sichuan University, Chengdu, China

**Keywords:** Method, Glucocorticoid resistance, Acute lymphoblastic leukemia, Hypoxia, Cell line

## Abstract

**Background:**

Drug-resistant cell lines, established from drug-sensitive cell lines by drug exposure in vitro, are the most useful cancer models in studies on the mechanism of chemoresistance. However, the success rate of the traditional approaches to construct such cell lines is low because a long time is required for the addition of drugs.

**Methods:**

A cell culture technique was used to establish the drug-resistant cell lines from their parental cells. Molecular and cellular biological techniques including flow cytometry, MTT assay, western blotting, and DNA fingerprinting analysis were used to characterize the drug-resistant cell lines. Nude mice were used for xenograft studies.

**Results:**

We established novel glucocorticoid (GC)-resistant cell lines from 3 GC-sensitive acute lymphoblastic leukemia (ALL) cell lines. First, we established a novel GC-resistant T-ALL cell line, CEM-C7/HDR, by mimicking the microenvironment of the bone marrow and culturing GC-sensitive CEM-C7–14 cells under hypoxia for 5 weeks with a single dexamethasone (Dex) treatment. The CEM-C7/HDR cells had been cultured continuously in drug-free medium under normoxia for 1 year. The IC_50_ and resistance index (RI) to Dex were maintained at 60~70 μM and 1500~1800, respectively, which is in consistent with the IC_50_ and RI of GC-resistant CEM-C1–15 cells. To clarify the reliability of the method, we subcloned CEM-C7–14 cells, and obtained Dex-resistant cell lines, CEM-C7-SC2/HDR and CEM-C7-SC14/HDR, from 2 monoclonal cells of CEM-C7–14 by the same method. Moreover, we obtained two additional Dex-resistant B-ALL cell lines, NALM-6/HDR and HXEX-ALL1/HDR, from NALM-6 and HXEX-ALL1 cells with the same approach.

**Conclusions:**

CEM-C7/HDR, NALM-6/HDR and HXEX-ALL1/HDR cell lines may serve as useful GC-resistant ALL models for both in vitro and in vivo studies. Culturing under hypoxic condition with a single Dex treatment is a novel and convenient approach for generating stable GC resistant cell lines.

**Electronic supplementary material:**

The online version of this article (10.1186/s13046-019-1280-2) contains supplementary material, which is available to authorized users.

## Background

Drug resistance remains a major challenge in cancer therapy. Although contemporary chemotherapy regimens have improved the long-term survival rate of childhood acute lymphoblastic leukemia (ALL) to nearly 90%, relapsed/refractory ALL is still a leading cause of tumor-related death in children [1]. The principal cause of treatment failure in relapsed/refractory ALL is chemoresistance, especially resistance to glucocorticoids (GCs) [2, 3]. Drug-resistant cell lines that are established from drug-sensitive cell lines are the most useful models in tumor research [4, 5]. The first in vitro-derived, drug-resistant cell line was reported by Biedler and Riehm in 1970 [6]. Drug-resistant cell lines have been used to investigate the mechanisms of drug resistance for nearly 50 years, but the methods used to construct such cell lines have remained essentially unchanged [4]. Two methods are available to acquire drug resistance in cell lines. One is stepwise dose-escalation continuous exposure, and the other is high-concentration pulsatile exposure. The stepwise escalation method has been used more commonly because it has a higher success rate [5]. Approaches that combine these two methods have also been successful at constructing drug-resistant cell lines. However, all such approaches took a long time (6~16 months, or more) [5]. Furthermore, the success rate for such approaches is low because a long time is required for the addition of drugs and cell recovery [4].

Drugs at a high concentration can kill all parental cells, which makes construction of a highly resistant cell line difficult. The resistance index (RI), defined as the ratio of the IC_50_ (drug concentration that inhibits 50% of cell growth) of the resistant cell line to that of its parental cell line, is approximately 2~80 in most cases [5]. The resistant phenotype may remain for several weeks or months after discontinuation of drug exposure; after that time, the cells have to be re-exposed to the drugs to regain resistance [4]. The time required to develop a resistant cell line and the phenotype obtained vary depending on the type of cell line and the selection agent being used [4]. The most important features of drug-resistant cell lines are those that conform to the clinical setting.

In a previous study, we observed the glycolytic phenotype in most drug-resistant leukemia cells, which is in concordance with the results of other studies [7]. Enhancement of the glycolytic phenotype may induce drug resistance [8]. ALL cells cultured under a hypoxic condition were more tolerant to chemotherapeutic drugs, including GCs [9]. Bone marrow (BM), which exists in a hypoxic microenvironment [10], is the most common site of relapse in ALL. Therefore, we attempted to simulate the clinical setting by establishing resistant cell lines under a hypoxic condition.

## Methods

### Cell lines and culture conditions

CEM-C7–14(GC sensitive) and CEM-C1–15 (GC resistant) cell lines were subcloned from the CCRF-CEM cell line [11], which was established from a patient with T-ALL [12]. The two cell lines were kindly provided by Dr. Thompson (The University of Texas Medical Branch). The GC-sensitive B-ALL cell line, NALM-6 was purchased from Shanghai Institute Cell Resources Bank; HXEX-ALL1 was established from a B-ALL patient by our laboratory. All cell lines were maintained in RPMI 1640 (HyClone, Logan, UT, USA) supplemented with 10% fetal bovine serum (FBS; HyClone) at 37 °C under a humidified atmosphere with 5% carbon dioxide (CO_2_) and 21% oxygen (O_2_; normoxic condition).

### Reagents and antibodies

Dexamethasone (Dex; Sigma-Aldrich, St. Louis, MO, USA) was dissolved in ethanol and used at a concentration of 0.1~ 0.5 μM. The final concentration of ethanol in the medium was 0.001%~ 0.005%, at which cell growth was not obviously altered. Propidium iodide (PI), trypan blue and 3-(4,5-dimethylthiazol-2-yl)-2,5-diphenyltetrazolium bromide (MTT) were purchased from Sigma. The Annexin V-PI Kit was purchased from *Roche* (Mannheim, Germany). Antibodies to Glut-1, HKII, LDH, p-LDH (Tyr10), 4E-BP1, p-4E-BP1 (Thr37/46), p70S6K, p-p70S6K (Thr389), AMPK, p-AMPK (Thr172), glucocorticoid receptor (GR), and p-GR (Ser211) were purchased from Cell Signaling Technology (Beverly, MA, USA). Horseradish peroxidase (HRP)–conjugated donkey anti-rabbit antibody and HRP-conjugated sheep anti-mouse antibodies were obtained from Santa Cruz Biotech (Santa Cruz, CA, USA). The β-Actin antibody was obtained from Kangchen Bio-Tech (Shanghai, China).

### Establishment of Dex-resistant ALL cell lines

Logarithmically growing cells were harvested and seeded in 6-well sterile plastic culture plates (Corning Inc., Corning, NY, USA) at a density of 1~3 × 10^4^/ml in RPMI-1640 medium supplemented with 10% FBS at 37 °C, and cultured in a tri-gas CO_2_ incubator (Thermo Fisher, Carlsbad, CA, USA) with a 5% CO_2_ and 1% O_2_ atmosphere (hypoxic condition). When the cell density reached 1~3 × 10^5^/ml, various concentrations of Dex (0.10 μM, 0.25 μM and 0.50 μM) were added to the culture plates. After 10~14 days in the lag-phase, the cells began to grow. The medium was replaced every 3~4 days to maintain the cells at a density of 5~10 × 10^5^/ml in the next 2~3 weeks. After 5~6 weeks of culture under the hypoxic condition, the cells were transferred to the normoxic condition and cultured in drug-free medium continuously for 1 year.

### Subcloning of ALL cells

Logarithmically growing cells were harvested and seeded in 6-well sterile plastic culture plates at a density of 5 × 10^2^/ml in methylcellulose RPMI-1640 medium containing 0.9% methylcellulose (MethoCult GFH4434; Sigma, St. Louis, MO, USA) and 10% FBS at 37 °C under a humidified atmosphere with 5% CO_2_ and 21% O_2_. Random aspiration of individual colonies growing in methylcellulose was undertaken on day 8 of the culture. Next, each colony was cultured in RPMI-1640 complete medium.

### Cell growth and viability assay

Cells were cultured in a 6-well sterile plastic culture plates at 1 × 10^5^/ml in RPMI-1640 medium with 10% FBS and grown for 7 days. Viable cells were counted using trypan blue staining every day. Doubling time (Td) was calculated for cells in exponential growth with the following equation: Td (h) = t × lg2/lg(N_t_/N_0_), where t is the time of continuous culture, N_t_ is the final number of cells, and N_0_ is the initial number of cells. Cell viability was evaluated by MTT assay. Briefly, cells were seeded in 96-well plates. Next, 0.5 mM MTT (final concentration) was added to each well for 4 h at 37 °C. Then, solubilization buffer (10% SDS in 0.01 M HCl) was added to each well, and the plates were further incubated for 24 h at 37 °C. The spectrophotometric absorbance was measured at 570 nm (reference 690 nm) using a multiplate reader (Multiskan Spectrum, Thermo Electron Co., Waltham, MA, USA). Values were obtained by comparing the experimental cells with their respective controls. Mean values were calculated from triplicate cultures.

### Chemosensitivity assays

The chemotherapeutic drugs, Dex, daunorubicin, vincristine, arabinoside, methotrexate and asparaginase, were purchased from Sigma. All cells were treated with increasing concentrations of different drugs for 48 h, followed by assessment of cell viability by MTT assay. The IC_50_ was calculated by linear interpolation.

### Cell cycle analysis

For each analysis, 10^6^ cells were harvested 48 h after treatment and fixed overnight in 70% ethanol at 4 °C. The cells were then washed and stained with 5 μg/ml PI in the presence of DNAse-free RNAse (Sigma). After 30 min at room temperature, the cells were analyzed via flow cytometry (Cytomics FC 500 and CXP & Multicycle software, Beckman Coulter Inc., Miami, FL, USA), acquiring 30,000 events.

### Immunophenotype analysis

For the detection of the immunophenotype of the CEM-C7/HDR and CEM-C7–14 cells, we used antibodies against the following targets: CD33, CD34, HLA-DR, cyTdT, cCD3, CD3, CD4, CD5, CD7, CD8, CD117, CD1α, CD2, CD10, CD19, CD20, CD13 and CD45 (Becton Dickinson Inc., Franklin Lakes, NJ, USA). Positivity for the antigens was determined using a FACSCalibur flow cytometer (Becton Dickinson Inc.).

### DNA fingerprinting analysis

The identity of the CEM-C1–15, CEM-C7–14, CEM-C7/HDR and CEM-C7/H cell lines was checked using DNA fingerprinting. DNA was prepared from these cells using the Qiagen DNeasy Blood Kit (Qiagen), according to the instructions provided by the manufacturer. The following 22 highly polymorphic short tandem repeat (STR) loci were tested by a multiplex PCR: *Amelogenin*, *CSF1PO*, *D13S317*, *D16S539*, *D5S818*, *D7S820*, *TH01*, *TPOX*, *vWA*, *Penta E*, *Penta D*, *D2S441*, *D2S1338*, *D3S1358*, *D6S1043*, *D8S1179*, *D10S1248*, *D12S391*, *D18S51*, *D19S433*, *D21S11* and *FGA*.

### Western blotting analysis

Briefly, cells (10^6^) were washed twice in cold PBS and then lysed by Laemmli sample buffer (Bio-Rad). Samples were boiled for 5 min at 100 °C. Proteins were separated by 8%~ 15% SDS–polyacrylamide gel electrophoresis and transferred onto nitrocellulose membranes (0.22 μm or 0.45 μm, Millipore). Non-specific binding sites were blocked with 5% non-fat dry milk dissolved in TBS (10 mM Tris-HCl, pH 7.6, 137 mM NaCl) with 0.1% Tween 20 (TTBS) for 2 h at room temperature, followed by incubation with primary antibody for 2 h at room temperature or at 4 °C overnight. The membranes were then washed 3 times in TTBS and incubated for 2 h at room temperature with secondary HRP–conjugated donkey anti-rabbit antibody or HRP-conjugated sheep anti-mouse antibody diluted 1:5000 in TTBS with 5% non-fat milk. Proteins were visualized by incubation with ECL plus (Millipore). All experiments were conducted independently at least 3 times. The level of the β-Actin protein was used as a control for the amount of protein loaded into each lane.

### Animal experiments

Cultured 5 × 10^6^ cells were subcutaneously injected into the right flanks of 6-week-old female BALB/c (nu/nu) nude mice, and 0.1 ml of PBS was injected into the left flanks as the control. Once palpable tumors were established, animals were randomized into 2 groups, each containing 4 mice. Mice were injected intraperitoneally daily with 15 mg/kg/d Dex (Dex group), or PBS (Control group) for 14 days. Tumor size was measured by calipers every 2 days. The approximate tumor volume was calculated using the eq. V = (length×width×depth)/2. All animals were ear-tagged and monitored individually throughout the experiment. All animal care was in compliance with the guidelines established by the internal Institutional Animal Care and Use Committee and Ethics Committee of Sichuan University. After the mice were euthanized, the tumor mass was excised, fixed in 10% formalin, and routinely processed for paraffin embedding. Five-millimeter-thick sections were obtained and prepared for standard histopathological examination.

### Statistical analysis

All assays were performed in triplicate, and data are expressed as mean values ± SD. One-way ANOVA was used to compare two groups. A *p*-value < 0.05 was considered to be significant.

## Results

### Establishment of Dex-resistant ALL cell lines

**Fig. 1:**
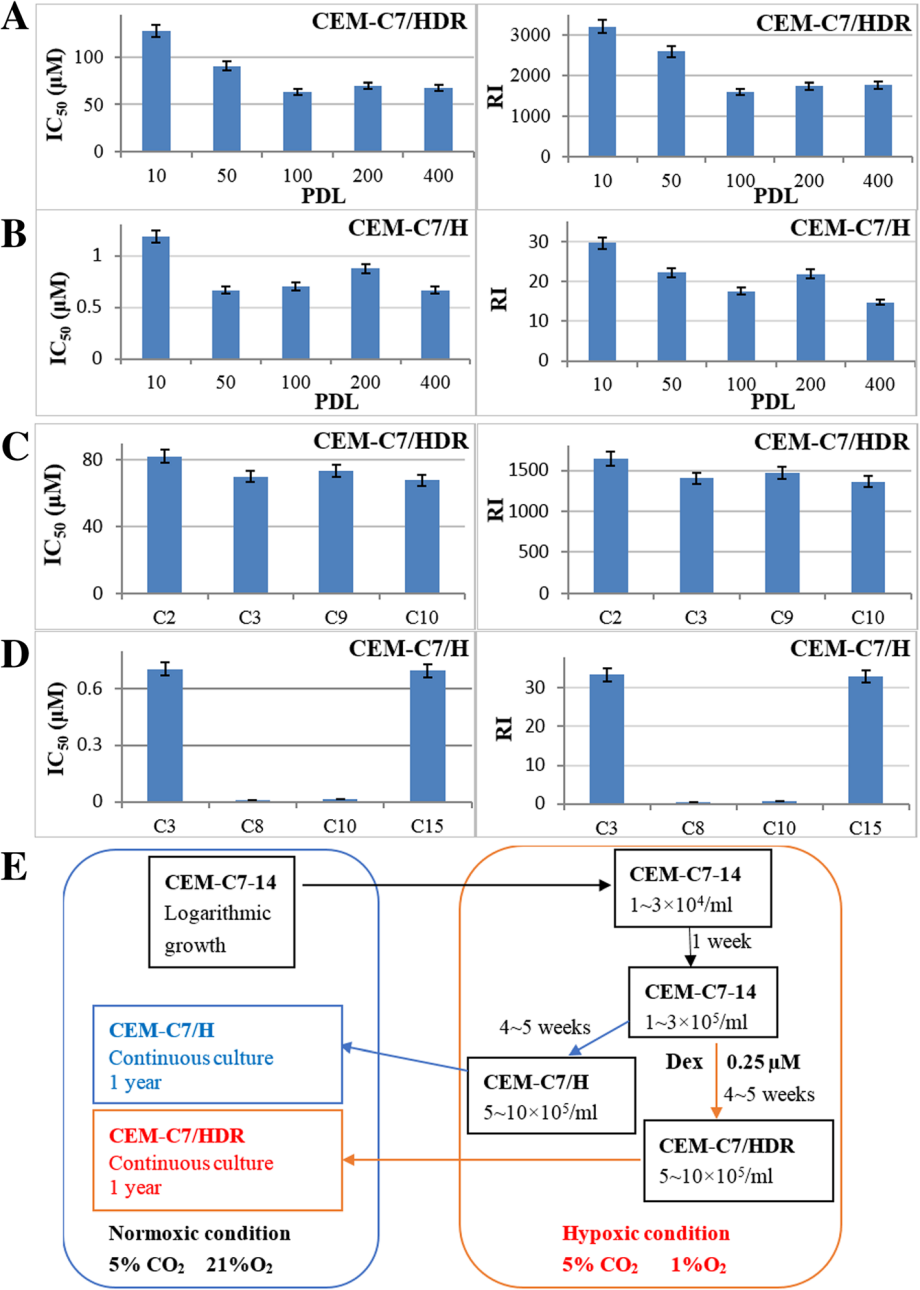
Resistance characteristics of CEM-C7/HDR and CEM-C7/H cell lines. **a** IC_50_ and RI of CEM-C7/HDR cells at 10~400 PDLs. Cells were cultured with increasing concentrations of Dex for 48 h. Cell viability was evaluated by MTT assays. The IC_50_ values were calculated by linear interpolation. Experiments were performed in triplicate. **b** IC_50_ and RI of CEM-C7/H cells at 10~400 PDLs. **c** IC_50_ and RI of subclones of CEM-C7/HDR cells. Four monoclones were screened. **d** IC_50_ and RI of subclones of CEM-C7/H cells. Four monoclones were screened. **e** Flow chart for constructing the GC-resistant cell lines

Logarithmically growing CEM-C7–14 cells were cultured under hypoxia condition for 5~6 weeks with or without dexamethasone (Dex) treatment. Then, the cells were transferred to the normoxic condition for 1 week. At that time, an MTT assay revealed that the IC_50_ values of the Dex groups, with a single treatment of 0.10 μM, 0.25 μM or 0.50 μM Dex respectively, were 50~150 μM at 48 h. We chose the 0.25 μM Dex group to be cultured continuously and constructed a stable resistant cell line, which was designated CEM-C7/HDR. The IC_50_ of Dex in the CEM-C7/HDR cells at a 10 cell population doubling level (PDL) was 128.1 ± 4.73 μM. Next, the IC_50_ and the RI of those cells decreased slowly, resulting in the PDL increasing from 10 to 100, where it stabilized after 100 PDL (Fig. 1a). After 1 year of Dex-free continuously culturing, the IC_50_ and RI were 60~70 μM and 1500~1800, respectively (Fig. 1a). Unexpectedly, the control group, which was cultured under the hypoxic condition with no Dex treatment, also gained Dex resistance. We named the control cell line CEM-C7/H, and its RI was 10~20 after 100 PDL (Fig. 1b). All CEM-C7/HDR subclones were resistant to Dex with RIs ranging from 1200 to 2000 (Fig. 1c). However, we obtained both resistant and sensitive subclones of CEM-C7/H, with RIs ranging from 0.3 to 33.4 (Fig. 1d). Interestingly, CEM-C7/H cells obtained cross-resistance to daunorubicin and vincristine, with RIs of 2.02 ± 0.23 and 1.71 ± 0.19, respectively. However, CEM-C7/HDR cells did not obtain cross-resistance to daunorubicin, vincristine, arabinoside, methotrexate or asparaginase. Figure 1e showed the procedure for constructing above cell lines. To clarify the mechanisms of resistance development, we selected two GC-sensitive CEM-C7–14 mono-clonal cells, named CEM-C7-SC2 and CEM-C7-SC14, with IC_50_ values of 0.04 μM and 0.06 μM, respectively. This method resulted in the development of two resistant cell lines named CEM-C7-SC2/HDR and CEM-C7-SC14/HDR, with IC_50_ values of 100~150 μM at 10PDL. The control groups, CEM-C7-SC2/H and CEM-C7-SC14/H, which were cultured under hypoxic condition with no Dex treatment, did not express the resistant phenotype. Additional file 1: Figure S1 showed the origin and name of the aforementioned cell lines.

To evaluate the repeatability of the method, we constructed stable Dex-resistant cell lines, NALM-6/HDR and HXEX-ALL1/HDR, from the NALM-6 and HXEX-ALL1 cell lines, using the same method. NALM-6/HDR and HXEX-ALL1/HDR were 28,000- and 4- fold more resistant to Dex than their parental cells (Additional file 2: Figure S2).

### Morphological and biological characteristics of CEM-C7/HDR cells

**Fig. 2:**
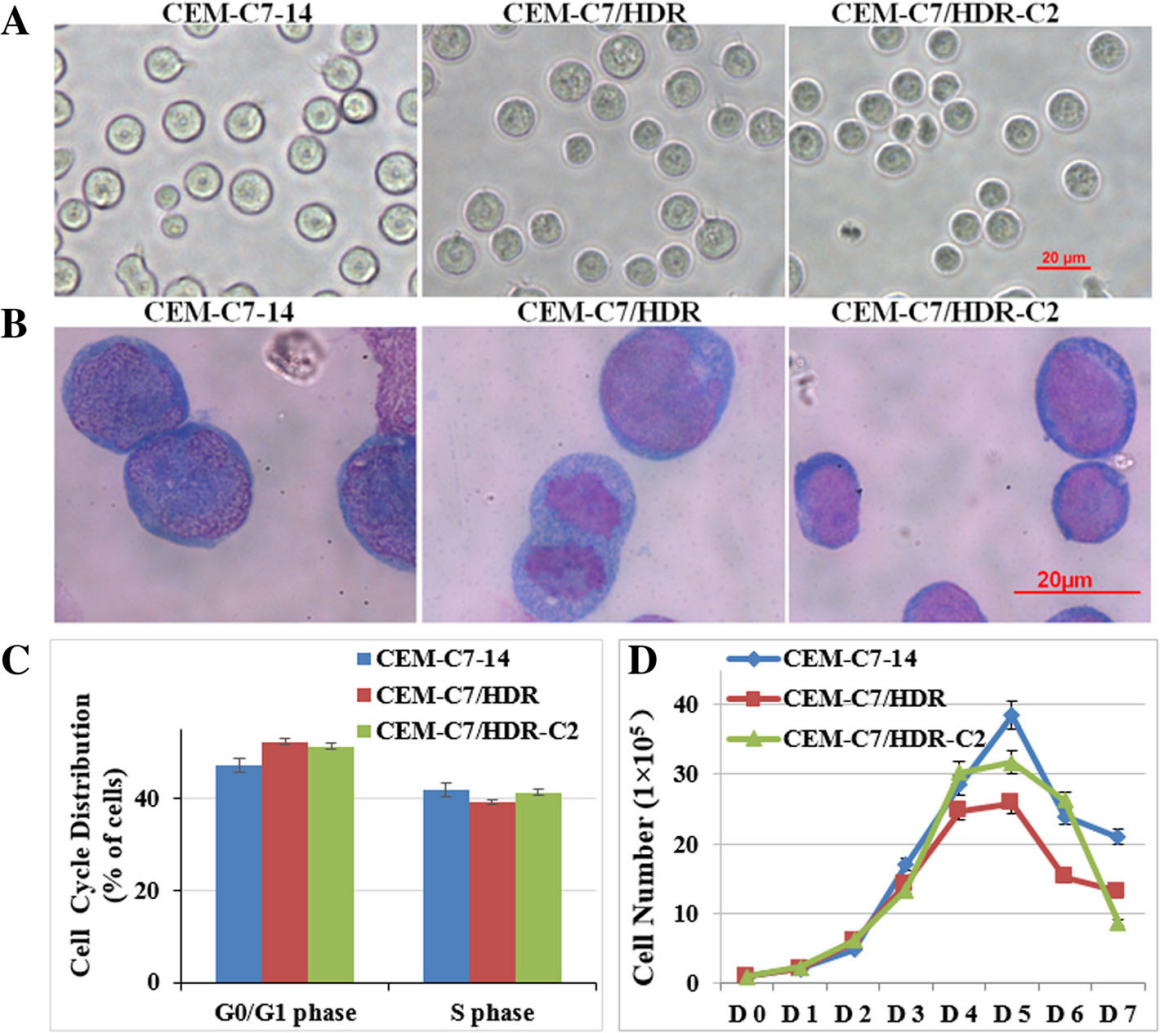
Morphological and biological characteristics of CEM-C7/HDR cell line. **a** Morphology of CEM-C7/HDR, CEM-C7/HDR-C2 and its parental CEM-C7–14 cells under phase contrast microscopy (× 400 magnification, scale bar 20 μM). **b** Wright-Giemsa staining of cells (× 1000 magnification, scale bar 20 μM). **c** Cell cycle distribution of CEM-C7/HDR, CEM-C7/HDR-C2 and CEM-C7–14 cells. Cells were cultured in a 6-well culture plate at 1 × 10^5^/ml, and cell cycle progression was analyzed by PI staining after 48 h. Experiments were performed in triplicate. The percentage of cells in the G_0_/G_1_ and S phase showed no significant difference (*P* > 0.05) compared with each other. **d** Growth curves of CEM-C7/HDR, CEM-C7/HDR-C2 and CEM-C7–14 cells. Cells were cultured in a 6-well culture plate at 1 × 10^5^/ml in RPMI-1640 medium with 10% FBS and grown for 7 days. Viable cells were counted using trypan blue staining every day. Experiments were performed in triplicate

**Fig. 3:**
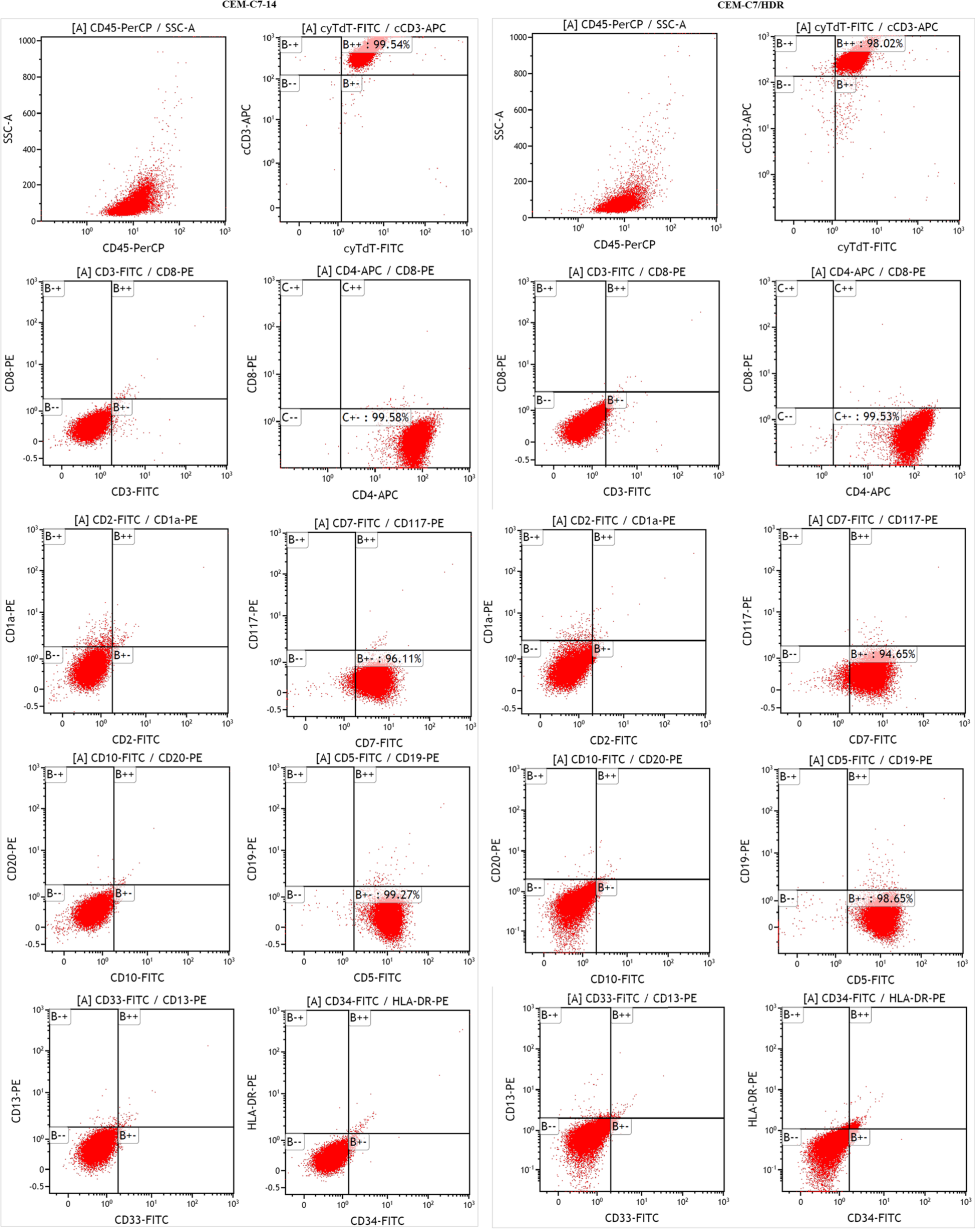
Immunophenotypic characteristics of CEM-C7/HDR cell line. The immunophenotype was analyzed using a FACSCalibur flow cytometer. CEM-C7/HDR and CEM-C7–14 displayed identical immunophenotype and were positive for cCD3, cyTDT, CD4, CD5 and CD7

The CEM-C7/HDR and CEM-C7/HDR-C2 (its monoclonal cell line) cells were grown in suspension as single cells, and Wright-Giemsa staining showed no obvious differences in morphology between the two resistant cell lines and the parental cell line, CEM-C7–14 (Fig. 2a and b). All three cells lines showed similar percentages of G_0_/G_1_ and S phase cells, with no statistically significant differences observed (*P* > 0.05) (Fig. 2c). They also exhibited similar growth curves (Fig. 2d). The three cell lines all stably proliferated in RPMI-1640 medium containing 10% FBS with population doubling times of 16~22 h. CEM-C7/HDR and CEM-C7–14 displayed identical immunophenotype with cCD3, cyTDT, CD4, CD5 and CD7 positive (Fig. 3). STR analysis confirmed that CEM-C7/HDR, CEM-C7/H, CEM-C7–14 and CEM-C1–15 were all derived from CCRF-CEM (Table 1).

**Table 1:** STR analysis of CEM-C1–15, CEM-C7–14, CEM-C7/HDR, CEM-C7/H and cells from the CEM-C7/HDR xenografts in nude mice

	CCRF-CEM^a^	CEM-C1–15	CEM-C7–14	CEM-C7/HDR	CEM-C7/H	CEM-C7/HDR xenograft
Amelogenin	X	X	X	X	X	X
CSF1PO	10, 11	11	11	10, 11	11	10, 11
D13S317	11, 12	11, 12	11, 12	11, 12	11, 12	11, 12
D16S539	10, 13	9, 13	10, 13	10, 13	10, 13	10, 13
D5S818	12, 13	12, 13	12, 13	12, 14	12, 13	12, 14
D7S820	9, 13	9, 13	9, 12	9, 11	9, 12	9, 11
TH01	6, 7	6, 7	6, 7	6, 7	6, 7	6, 7
TPOX	8	8	8	8	8	8
vWA	17, 19	17, 18	17, 19	17, 19	17, 19	17, 19
Penta E	NO	5, 15	5, 14	5, 14	5, 14	5, 14
Penta D	NO	11	11	11	11	11
D2S441	NO	10, 14	11, 14	11, 14	11, 14	11, 14
D2S1338	NO	23, 24	24, 25	24, 25	24, 25	24, 25
D3S1358	NO	14, 15	15	15	15	15
D6S1043	NO	11, 14	11, 14	11, 14	11, 14	11, 14
D8S1179	NO	13	13	13	13	13
D10S1248	NO	13, 16	13, 14, 15	13, 16	13, 14, 15	13, 16
D12S391	NO	17, 21	18, 19, 20	18, 20	18, 19, 20	18, 20
D18S51	NO	12, 18	13, 17	13, 17	13, 17	13, 17
D19S433	NO	14, 16	14, 15	14, 15	14, 15	14, 15
D21S11	NO	30, 33.2	30, 33.2	30, 33.2	30, 33.2	30, 33.2
FGA	NO	23	23	23	23	23

^a^: STR profile of CCRF-CEM was extracted from the database of ATCC cell bank

### Expression of proteins associated with GC resistance and hypoxia in CEM-C7/HDR

**Fig. 4:**
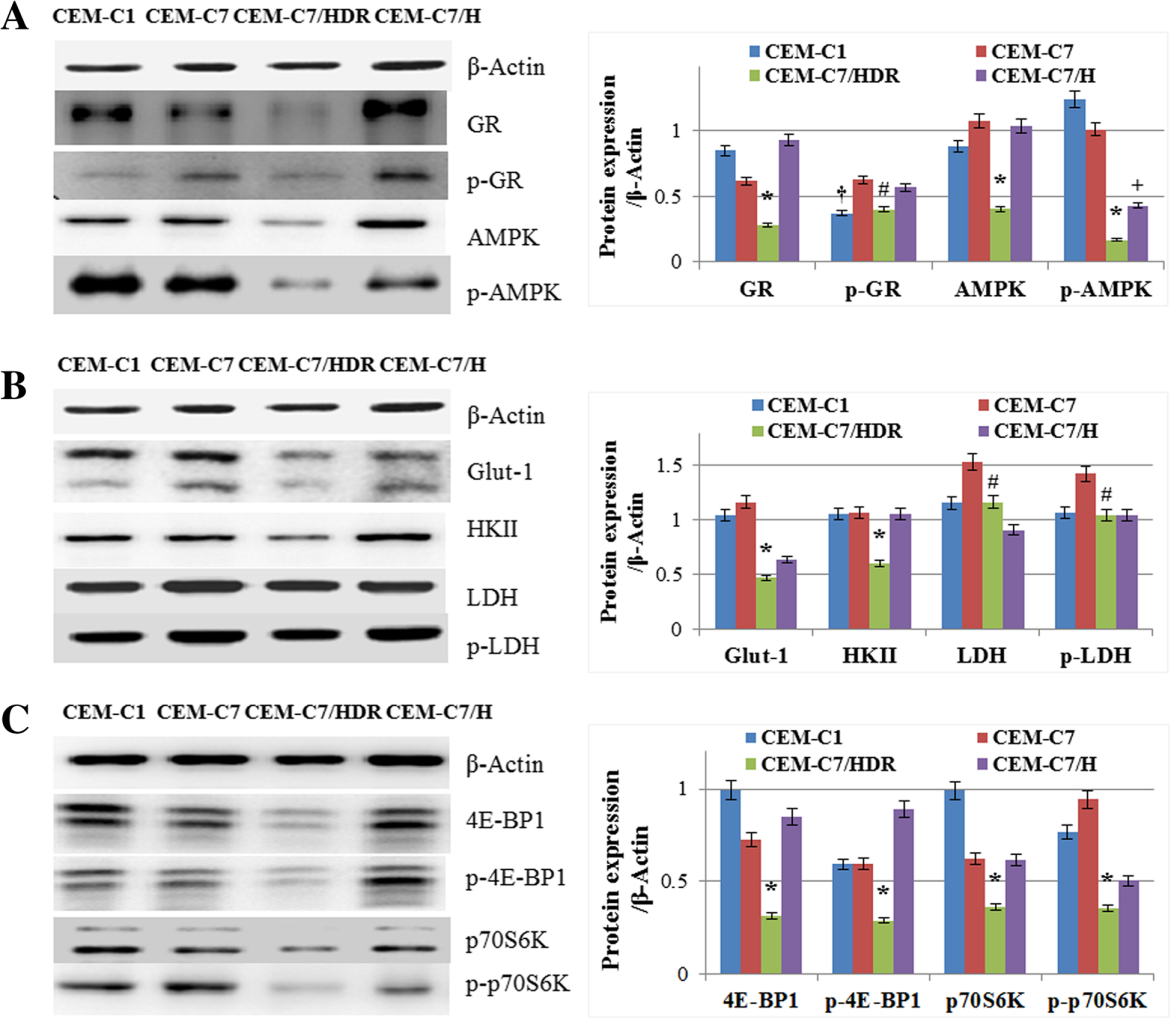
Expression of GC-resistance- and hypoxia-associated proteins in CEM-C7/HDR. **a** Cells were lysed, and extracts were analyzed by western blotting for GR, p-GR (Ser211), AMPK and p-AMPK (Thr172). β-Actin was used as an internal control. Bar graphs show the ratio of proteins to β-Actin. For all experiments, values of triplicate experiments are shown as the mean ± SD. *: *p* < 0.01 versus CEM-C1–15, CEM-C7–14 and CEM-C7/H. #: *p* < 0.01 versus CEM-C7–14 and CEM-C7/H. †: *p* < 0.01 versus CEM-C7–14 and CEM-C7/H. +: *p* < 0.01 versus CEM-C1–15 and CEM-C7–14. **b** Western blot analysis of Glut-1, HKII, LDH and p-LDH (Tyr10). β-Actin was used as an internal control. Bar graphs show the ratio of protein to β-Actin. *: *p* < 0.01 versus CEM-C1–15, CEM-C7–14 and CEM-C7/H. #: *p* < 0.01 versus CEM-C7–14. **c** Western blot analysis of 4E-BP1, p-4E-BP1 (Thr37/46), p70S6K and p-p70S6K (Thr389). β-Actin was used as an internal control. Bar graphs show the ratio of protein to β-Actin. *: *p* < 0.01 versus CEM-C1–15, CEM-C7–14 and CEM-C7/H

Low expression of GR may be a major cause of GC resistance in leukemia cells [13, 14]. As shown in Fig. 4a, the expression of GR and p-GR (Ser211) were significantly lower in the CEM-C7/HDR cells than in the CEM-C7–14 and CEM-C7/H cells. In GC resistant cells, GR expression was lower in CEM-C7/HDR than in CEM-C1–15 (*p* < 0.01), the p-GR (Ser211) expression showed no significant difference between the two cell lines (*p* > 0.05). We obtained CEM-C7/HDR and CEM-C7/H cell lines from culturing under hypoxia condition. Therefore, we detected the expression of the energy sensor proteins, AMPK and p-AMPK. However, the expression of AMPK and p-AMPK in CEM-C7/HDR were both lower than in CEM-C7–14 and CEM-C1–15 (*p* < 0.01) (Fig. 4a). In CEM-C7/H, the expression of p-AMPK was lower than in CEM-C7–14, although the AMPK expression showed no significant difference (*p* > 0.05) (Fig. 4a). It is generally known that culturing under hypoxic conditions may induce a cellular switch in energy production, which is characterized by an increase in glycolysis phenotype in cancer cells [15]. In our study, although the CEM-C7/HDR cell line was cultured in hypoxic condition for 5 weeks, the expression of glycolysis-associated proteins, Glut-1, HKII, LDH and p-LDH, were all lower than that in CEM-C7–14 (*p* < 0.01) (Fig. 4b). Dysregulated activation of PI3K-Akt-mTOR pathway may relate to the chemotherapeutic resistance, including GC resistance, in hematological malignances [16, 17]. However, in CEM-C7/HDR, the expression of the two main downstream effectors of mTOR, 4E-BP1 and p70S6K, and their phosphorylated status p-4E-BP1 (Thr37/46) and p-p70S6K (Thr389), were all obviously lower than CEM-C7–14, CEM-C7/H and CEM-C1–15 cells (p < 0.01) (Fig. 4c). Thus, the molecular mechanisms of GC resistance in the CEM-C7/HDR cell line should be far different from that in the CEM-C1–15 cells.

### The tumorigenicity of CEM-C7/HDR cells

**Fig. 5:**
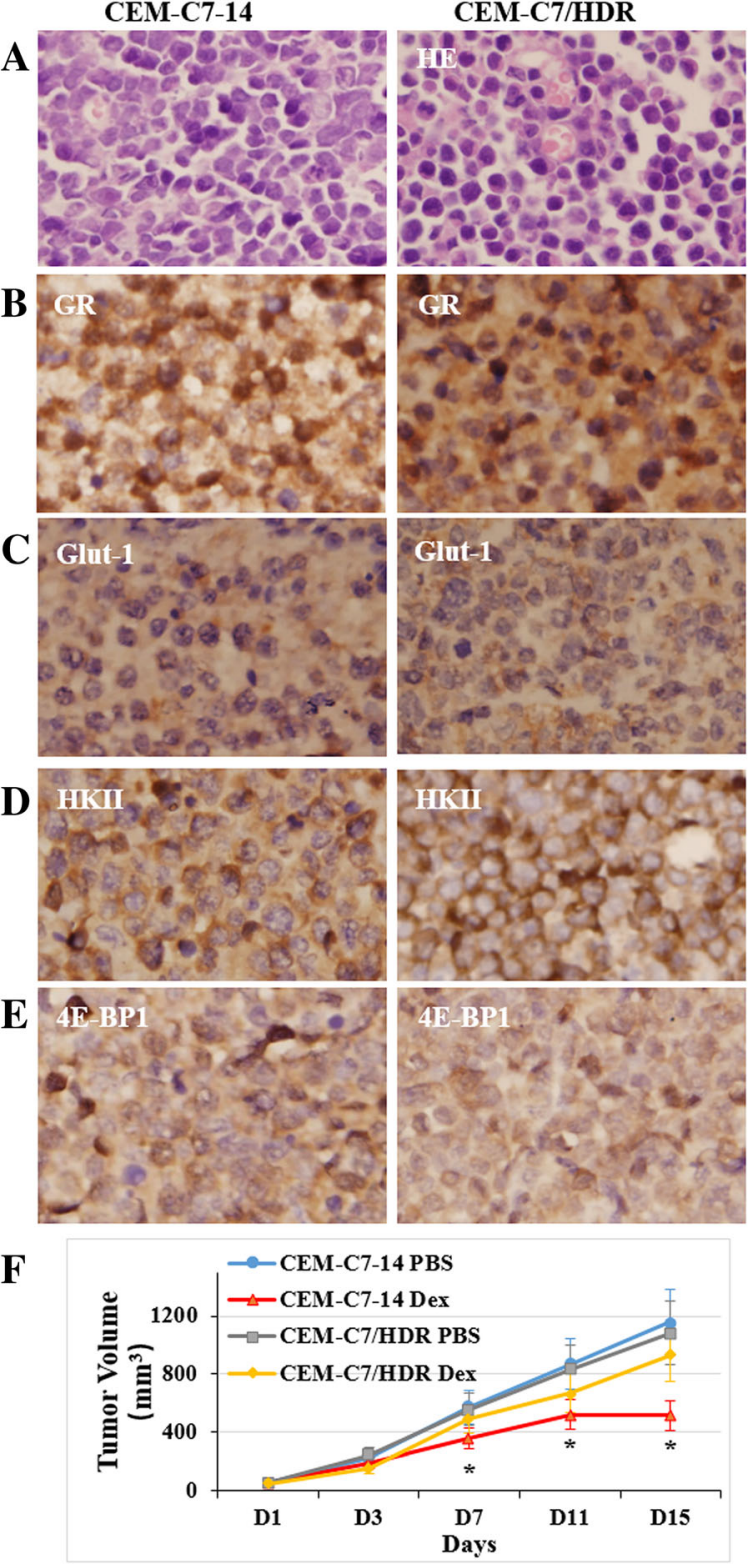
Tumorigenicity of CEM-C7/HDR cells. **a** HE staining of the tumors derived from CEM-C7–14 and CEM-C7/HDR showed that the xenografts were composed of leukemia cells and blood vessels. Original magnification: × 400 magnification. **b**-**e** On IHC staining, both tumors derived from CEM-C7–14 and CEM-C7/HDR expressed GR, Glut-1, HKII, and 4E-BP1 proteins with a similar pattern, respectively. Original magnification: × 400 magnification. **f** CEM-C7/HDR or CEM-C7–14 cells were subcutaneously inoculated into BALB/C nude female mice. Once palpable tumors were established, animals were treated with 15 mg/kg/d Dex (Dex group), or PBS (Control group) for 14 days. Tumor volume was measured every 2 days, and data are expressed as mean volume ± SD (*n* = 4 tumors per group). Dex treatment had little effect on CEM-C7/HDR xenografts in nude mice, whereas inhibited tumor growth significantly in CEM-C7–14 xenografts mice. *: *p* < 0.05 versus CEM-C7–14 PBS group, CEM-C7/HDR PBS and Dex group

In an in vivo transplantation test, subcutaneous injection of CEM-C7–14 and CEM-C7/HDR resulted in the development of tumors in all 12 mice (100%). After 20 days, the mean volume of the tumors generated by subcutaneous injection with CEM-C7–14 and CEM-C7/HDR were 717.8 ± 309.6 mm^3^ (*n* = 6) and 803.0 ± 294.9 mm^3^ (*n* = 6), respectively. Hematoxylin and eosin (HE) staining indicated that the tumor masses were composed of leukemia cells (Fig. 5a). Immunohistochemistry (IHC) staining showed that the expression locations of GR, Glut-1, HKII, and 4E-BP1 proteins were consistent in the xenografts of CEM-C7–14 and CEM-C7/HDR (Fig. 5b-e). The results from STR analysis indicated that subcutaneous tumors derived from the corresponding CCRF-CEM cells (Table 1).

Having shown the GC-resistant phenotype of CEM-C7/HDR in vitro, we examined the in vivo efficacy of Dex given intraperitoneally in CEM-C7/HDR and CEM-C7–14 xenografts in nude mice. As anticipated, 15 mg/kg/d Dex used alone showed almost no antitumor effect (*p* > 0.05) in CEM-C7/HDR xenografts mice, whereas inhibited tumor growth obviously in CEM-C7–14 xenografts mice (*p* < 0.05, Fig. 5f).

## Discussion

Preclinical studies on cancer cell lines have played an important role in our understanding of cancer biology and high-throughput screening for drug development. Molecular and cellular changes that are observed in a drug-resistant cell line, when compared to its drug-sensitive counterpart, can suggest molecular mechanisms underlying drug resistance, and locus of drug action is inferred by the presence of those particular alterations. There is a controversy regarding construction of drug-resistant cell lines by the traditional drug-exposure method and whether or not they can reflect the clinical setting [4]. We should establish a method that matches the manner in which chemotherapy is administered to patients.

GCs specifically induce apoptosis in malignant lymphoblasts and are thus pivotal in the treatment of lymphoid malignancies, especially ALL [18]. However, GC resistance is a therapeutic problem that accounts for most of the treatment failures [2, 3]. The exact molecular mechanism remains poorly understood. The hypoxic microenvironment of BM promoted ALL cells survival and conferred resistance to chemotherapy [9, 19, 20]. Hence, we aimed to construct Dex-resistant cell lines under conditions that mimic ALL cells growing under the hypoxic BM microenvironment. First, GC sensitive ALL cells were cultured under a hypoxic environment for approximately 1 week to accommodate the hypoxic condition. Second, we added a therapeutic dose of Dex according to the clinical chemotherapy protocol to the parental cells and cultured the cells under the hypoxic condition for 4~5 weeks. Finally, the cells were transferred to the normoxic condition to mimic the clinical setting, similar to how ALL cells are released from BM into the peripheral blood. To our delight, this strategy works. Using this method, we obtained Dex-resistant ALL cell lines from GC sensitive parental cell lines, CEM-C7–14, NALM-6, HXEX-ALL1 and their subcloned cell lines. Furthermore, the resistant phenotype acquired by this approach was stable after 1 year of continuous culture in Dex-free medium.

The established CEM-C7/HDR cells were almost identical with its parental cells, CEM-C7–14, with respect to morphology, cell proliferation, STR and immunophenotype characteristics. In addition, CEM-C7/HDR cells showed a high tumorigenic capacity similar to CEM-C7–14, and the expression location of proteins associated with GC resistance and hypoxia, GR, Glut-1, HKII, and 4E-BP1, were consistent in the xenografts of CEM-C7–14 and CEM-C7/HDR. The resistance characteristics of CEM-C7/HDR cells were similar to that of CEM-C1–15 cells. The IC_50_ of Dex in CEM-C1–15 cells was 61.71 ± 4.25 μM, and the RI for these cells was 1620 ± 128. The IC_50_ values of Dex in other GC-resistant leukemia-lymphoma cell lines, such as Jurkat, Raji, Molt-4 and MV4–11, ranged from 30 to 90 μM. Therefore, the GC-resistant cell line CEM-C7/HDR, which was constructed by mimicking the in vivo microenvironment of BM, does resemble the clinic setting to some extent.

It is generally accepted that GCs induce apoptosis in lymphoid blast cells through activation of the GR, and low expression of GR may contribute to GC resistance in leukemia cells [13, 14]. As anticipated, our results showed that CEM-C7/HDR exhibited an obviously lower expression of GR and p-GR (Ser211) compared with its parental cells CEM-C7–14, and p-GR (Ser211) was low expressed in both two GC-resistant cell line, CEM-C7/HDR and CEM-C1–15. Therefore, low expression of p-GR (Ser211) might be the main cause for GC resistance in T-ALL, which needs further investigation in T-ALL patients.

Tumor cells respond to hypoxic stress by upregulating various genes involved in glucose uptake, glycolysis, and angiogenesis, all of which are essential in maintaining nutrient availability and intracellular ATP levels [21, 22]. AMPK is an energy sensor that is pivotal in maintaining cell metabolic homeostasis [23, 24]. Metabolic stresses, such as glucose starvation and hypoxia, activate AMPK via threonine 172 phosphorylation by AMPK upstream kinases [23, 24]. AMPK is essential for T-ALL cell survival and disease progression [25]. Surprisingly, the expression of p-AMPK was lower in CEM-C7/HDR and CEM-C7/H than in CEM-C7–14 and CEM-C1–15. Moreover, the expression of AMPK decreased also in CEM-C7/HDR cells. It is reported that AMPK plays a role in regulating growth and survival of multiple cancer cells including leukemia cells [26, 27]. Conversely, other studies showed that activation of AMPK may suppress the growth of some tumors including T-ALL [28–30]. A recent paper reported that levels of p-AMPK and AMPKα1 mRNA are dramatically decreased in clinical leukemia cells, which might play a role in drug resistance in leukemia [31]. Although AMPK plays a paradoxical dual role in ALL survival and progression [25, 32], decreased expression of AMPK and p-AMPK might contribute to GC resistance in CEM-C7/HDR.

Hypoxic stress can induce glycolysis and the mTOR signaling pathway, which would contribute to chemoresistance [9, 20, 21, 33]. Moreover, loss of AMPK can induce glycolysis and mTOR pathway also [24, 29, 32]. Unexpectedly, in CEM-C7/HDR, key enzymes of glycolysis, Glut-1, HKII, LDH and p-LDH, were all decreased compared to that in its parental cell line CEM-C7–14. Similarly, the mTOR signaling pathway was surprisingly depressed in CEM-C7/HDR. It is intriguing that while AMPK functions as an antagonist of mTOR, both AMPK and mTOR signaling pathways were depressed in CEM-C7/HDR cells; this result suggested additional unclear regulation mechanisms, which deserves further investigation. A recent study compared overall survival in 2 subsets of patients with high and low PI3K-Akt-mTOR activation in 77 AML patients and showed no obvious difference [34]. An in vitro test showed that an AMPK activator and mTOR inhibitor, such as metformin and rapamycin, can effectively inhibit the growth of ALL cells [29, 35]. However, those drugs showed a clinically relevant response only in a minority of patients [34, 36]. Some ALL cells displayed poor response to mTOR inhibition [37]. In addition, other studies showed that long-lasting (chronic) hypoxia may inhibits mTOR pathway through multiple pathways, including BNIP3, PML, AMPK, and REDD1, to promote hypoxic tolerance [38]. Therefore, the relative low expression of AMPK and mTOR pathway might be another key point to understand the mechanisms of GC resistance, and CEM-C7/HDR may provide a novel model that represents a subset of GC-resistant ALL cells.

Resistant cell lines can be established by drug exposure and induction of extrinsic mutations or by selection of clones based on the strength of an intrinsic mutation [5]. CEM-C7/H attained a resistant phenotype by culturing under hypoxia condition for 5 weeks without Dex treatment. However, it is noteworthy that only part of subclones of CEM-C7/H attained Dex resistance. Moreover, NALM-6, HXEX-ALL1 and subclones of the three cell lines did not attained a resistant phenotype by only hypoxic culture. Therefore, hypoxia may act as a compressive stress that causes resistant clones to become dominant in the culture. In other words, hypoxia stress could not select out resistant clones from cells without intrinsic resistance-related mutations. Interestingly, CEM-C7/H cells obtained cross-resistance to daunorubicin and vincristine. However, CEM-C7/HDR cells did not obtain cross-resistance to daunorubicin, vincristine, arabinoside, methotrexate or asparaginase. In general, culturing under hypoxia condition with a single Dex treatment is a novel and convenient approach for generating stable GC resistant cell lines. The protocol presented here should be modified for use in establishing cell lines that are resistant to other drugs and for identifying molecular inhibitors that can target cancer cells living in a hypoxic environment. Moreover, in leukemia cells, a hypoxic condition may act as a selection pressure to develop resistant clones.

## Conclusions

Here, we constructed a new method to establish GC-resistant leukemia cell lines. These newly established resistant cell lines may serve as valuable in vitro and in vivo tools for further investigation on the potential mechanisms of GC resistance, especially the role of the hypoxic microenvironment in GC resistance.

## Additional files

Additional file 1: **Figure S1.** Cell lines originated from CCRF-CEM. (A) CEM-C7-14 and CEM-C1-15 cell lines were subcloned from CCRF-CEM. (B) CEM-C7-SC2 and CEM-C7-SC14 were subcloned from CEM-C7-14. After culturing under hypoxia for 5 weeks with or without Dex, CEM-C7-SC2/HDR, CEM-C7-SC14/HDR, CEM-C7-SC2/H, and CEM-C7-SC14/H were constructed. (C) After culturing CEM-C7-14 under hypoxia for 5 weeks with or without Dex, CEM-C7/HDR and CEM-C7/H were constructed. CEM-C7/HDR were subcloned into CEM-C7/HDR-C2, CEM-C7/HDR-C3, CEM-C7/HDR-C9, and CEM-C7/HDR-C10. CEM-C7/H were subcloned into GC-resistant CEM-C7/H-C3 and CEM-C7/HDR-C15, and GC-sensitive CEM-C7/H-C8 and CEM-C7/H-C10. (TIF 294 kb)

Additional file 2: **Figure S2.** Resistance characteristics of NALM-6/HDR and HXEX-ALL1/HDR cell lines. (A) IC_50_ and RI of NALM-6/HDR cells at 10~400 PDLs. (B) IC_50_ and RI of HXEX-ALL1/HDR cells at 10~100 PDLs. Cells were cultured with increasing concentrations of Dex for 48 h. Cell viability was evaluated by MTT assays. The IC_50_ values were calculated by linear interpolation. Experiments were performed in triplicate. (TIF 165 kb)

## Data Availability

The datasets used and analyzed during the current study are available from the corresponding author on a reasonable request.

## Abbreviations

ALL: Acute lymphoblastic leukemia
BM: Bone marrow
Dex: Dexamethasone
GC: Glucocorticoid
PDL: Population doubling level
RI: Resistance index
Td: Doubling time
