# Supplementary figures and images for: A novel method to establish glucocorticoid resistant acute lymphoblastic leukemia cell lines
Source: J Exp Clin Cancer Res. 2019 Jun 20;38:269. doi: 10.1186/s13046-019-1280-2 (PMC6585113; doi:10.1186/s13046-019-1280-2)

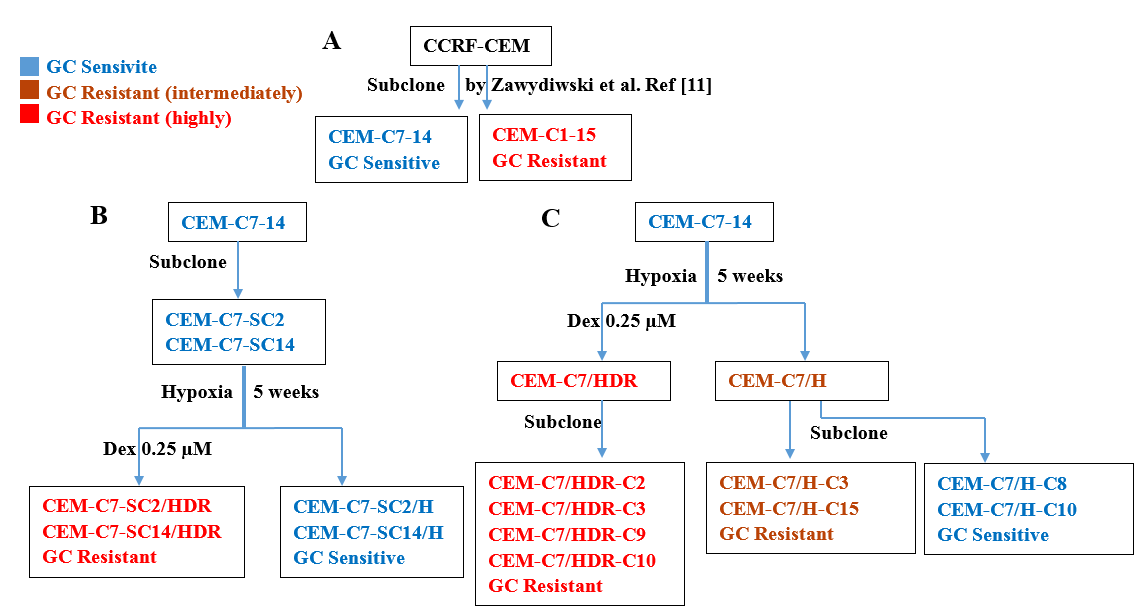

Supplement: Supplementary file 1 — Figure S1. Cell lines originated from CCRF-CEM. (A) CEM-C7-14 and CEM-C1-15 cell lines were subcloned from CCRF-CEM. (B) CEM-C7-SC2 and CEM-C7-SC14 were subcloned from CEM-C7-14. After culturing under hypoxia for 5 weeks with or without Dex, CEM-C7-SC2/HDR, CEM-C7-SC14/HDR, CEM-C7-SC2/H, and CEM-C7-SC14/H were constructed. (C) After culturing CEM-C7-14 under hypoxia for 5 weeks with or without Dex, CEM-C7/HDR and CEM-C7/H were constructed. CEM-C7/HDR were subcloned into CEM-C7/HDR-C2, CEM-C7/HDR-C3, CEM-C7/HDR-C9, and CEM-C7/HDR-C10. CEM-C7/H were subcloned into GC-resistant CEM-C7/H-C3 and CEM-C7/HDR-C15, and GC-sensitive CEM-C7/H-C8 and CEM-C7/H-C10. (TIF 294 kb) [file 13046_2019_1280_MOESM1_ESM.tif]

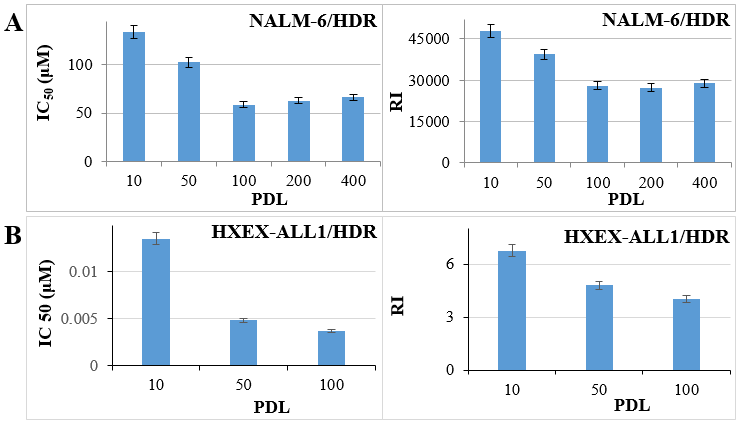

Supplement: Supplementary file 2 — Figure S2. Resistance characteristics of NALM-6/HDR and HXEX-ALL1/HDR cell lines. (A) IC50 and RI of NALM-6/HDR cells at 10~400 PDLs. (B) IC50 and RI of HXEX-ALL1/HDR cells at 10~100 PDLs. Cells were cultured with increasing concentrations of Dex for 48 h. Cell viability was evaluated by MTT assays. The IC50 values were calculated by linear interpolation. Experiments were performed in triplicate. (TIF 165 kb) [file 13046_2019_1280_MOESM2_ESM.tif]
